# Genomic characterization of a Brazilian multidrug-resistant *Klebsiella pneumoniae* ST17 isolate associated with bovine mastitis

**DOI:** 10.1007/s11259-026-11622-5

**Published:** 2026-11-11

**Authors:** Guilherme Castro Luz da Silva, Rossiane de Moura Souza, Ana Carolina Paulo Vicente, Sergio Mascarenhas Morgado, Juliana Souza Alves, Sílvia Conceição Reis Pereira Mello, Gustavo Luis de Paiva Anciens Ramos, Alice Gonçalves Martins Gonzalez

**Affiliations:** 1 Faculty of Pharmacy, Department of Food Science, Hygiene and Food Microbiology Laboratory, Fluminense Federal University, Niterói, 24241-000 Dr. Mário Vianna Street, 523, Brazil; 2 State Centre for Research in Animal Health, Biotechnology Laboratory, Agricultural Research Company of the State of Rio de Janeiro, Rio de Janeiro, 24120-191 Brazil; 3 Oswaldo Cruz Institute - FIOCRUZ, Rio de Janeiro, 21040-360 Brazil; 4 Department of Natural Products and Foods, Federal University of Rio de Janeiro, Rio de Janeiro, 21941-902 Brazil

**Keywords:** Resistome, Virulome, extended-spectrum β-lactamase, Iron acquisition, Whole-genome sequencing, One Health

## Abstract

This study presents the genomic and phenotypic characterization of a multidrug-resistant *Klebsiella pneumoniae* (MDR-Kp) isolate (LC808) obtained from a case of bovine mastitis in Brazil. Antimicrobial susceptibility testing revealed an MDR phenotype, indicating resistance to penicillins, cephalosporins, monobactams, aminoglycosides, tetracyclines, fluoroquinolones, and trimethoprim-sulfamethoxazole. The presence of an extended-spectrum β-lactamase (ESBL) was also confirmed. Whole-genome sequencing identified the isolate as sequence type 17 (ST17) and revealed the presence of the KL127 and O13 loci. The resistome contained genes associated with antimicrobial resistance, including *bla*_CTX−M−15_, *bla*_TEM−1_, *bla*_SHV−187_, and *bla*_OXA−1_ (β-lactams); *aph(6)-Id*, *aph(3’’)-Ib*, *aph(3’)-Ia*, *aac(3)-IIe*, and *aac(6’)-Ib10* (aminoglycosides); *tet(A)* (tetracyclines); *qnrB17* (fluoroquinolones); and *sul2* (sulfonamides). A class 1 integron carrying *dfrA14* was also identified. The virulome included type 1 and type 3 fimbriae, a type VI secretion system, and multiple iron-acquisition systems (*fecABCDEIR*). Identifying this clinically relevant lineage that carries multiple resistance and virulence determinants highlights the presence of MDR-Kp in bovine mastitis in Brazil. These findings provide the necessary genomic baseline data to support ongoing genomic surveillance of antimicrobial-resistant bacteria in dairy herds within a One Health framework.

**Supplementary Information:**

The online version contains supplementary material available at https://doi.org/10.1007/s11259-026-11622-5.

## Introduction

Bovine mastitis is one of the diseases with the greatest economic impact on the global dairy industry (Rasmussen et al. 2024). Beyond production losses, antimicrobial-resistant bacteria are a major concern within the One Health framework due to the potential circulation of bacterial strains and resistance determinants among animals, humans, food, and the environment (Alves et al. 2024). In this context, *Klebsiella pneumoniae* is notable for its ability to acquire and disseminate clinically relevant resistance and virulence genes (Benavides et al. 2026). Although specific sequence types (STs), such as ST17, are often associated with multidrug-resistant human clinical infections, sequence type alone does not imply equivalent pathogenicity or epidemiological risk. This is because the clinical impact is heavily influenced by the accessory genome (Bahati et al., 2025).

To establish transmission dynamics, sampling must encompass human, animal, and environmental interfaces. However, characterizing multidrug-resistant *K. pneumoniae* lineages isolated from cases of clinical mastitis can provide essential data on the resistome circulating in dairy herds. Therefore, an important first step in genomic surveillance in veterinary settings is combining phenotypic susceptibility testing with high-resolution whole-genome sequencing (WGS) of clinical isolates (Wang et al. 2025).

Despite advances in the genomic epidemiology of *K. pneumoniae*, significant gaps remain in our understanding of the occurrence and resistome composition of high-risk strains associated with bovine mastitis in South America. In Brazil, for instance, *K. pneumoniae* ST17 isolates carrying extended-spectrum β-lactamase (ESBL) determinants have been documented in human clinical settings (Andrade et al. 2018). However, genomic data on the prevalence of ESBL-producing ST17 strains in livestock is limited. To address this gap, this study used whole-genome sequencing to perform a detailed genomic and phenotypic characterization of an ESBL-producing, multidrug-resistant *K. pneumoniae* ST17 isolate (LC808), which was recovered from a cow with clinical mastitis on a family farm in Rio de Janeiro, Brazil, to address this gap.

## Materials and methods

### Isolation and identification

The *K. pneumoniae* strain LC808 is part of the culture collection of the Rio de Janeiro State Agricultural Research Corporation (PESAGRO-RIO). The bacterium was first isolated in August 2022 from a sample of raw milk collected during a routine veterinary examination of a lactating cow with clinical mastitis at a small, family-owned dairy farm in Rio de Janeiro, Brazil. Prior to sample collection, the animal had not received any antimicrobial treatment (Supplementary Table S1). A raw milk sample corresponding to one mammary quarter was aseptically collected from the affected right hind quarter. The sample was transported under refrigeration (4–8 °C) to the PESAGRO-RIO biotechnology laboratory within 48 h. Primary bacterial isolation was performed by diluting the milk sample in buffered peptone water (HiMedia^®^, India) supplemented with Tween 20 and plating it on a chromogenic medium (Compact Dry™ EC, Nissui Pharmaceutical Co., Tokyo, Japan) according to the manufacturer’s instructions. Identification at the species level was confirmed by matrix-assisted laser desorption / ionization time-of-flight mass spectrometry (MALDI-TOF MS; Microflex LT, Bruker Daltonics; score = 2.370). The archived isolate was subsequently evaluated for antimicrobial susceptibility and confirmed as an ESBL producer. Based on its confirmed MDR phenotype and ESBL production, strain LC808 underwent WGS for in-depth genomic characterization.

### Antimicrobial susceptibility testing (AST)

Antimicrobial susceptibility testing was performed using the Kirby-Bauer disk diffusion method according to Clinical and Laboratory Standards Institute (CLSI, 2021). Pure overnight colonies were suspended in sterile 0.85% saline until reaching the 0.5 McFarland standard, then swabbed onto Mueller-Hinton agar (MHA; HiMedia^®^, India). Antimicrobial disks (Sensifar^®^, Brazil) were placed on the plates, which were then incubated aerobically at 35 ± 2 °C for 16–18 h (Supplementary Table S2). *Escherichia coli* ATCC 25,922 was used as the quality control strain.

Intermediate and resistant profiles were categorized as non-susceptible (Magiorakos et al. 2012). The MDR phenotype was defined as non-susceptibility to at least three antimicrobial classes. ESBL production was confirmed using the phenotypic combined disk test with ceftazidime and cefotaxime, both alone and in combination with clavulanic acid (CLSI, 2021).

### Whole-genome sequencing (WGS) and genomic analysis

Genomic DNA was extracted using the NucleoSpin Microbial DNA kit (Macherey-Nagel) and the sequencing library was prepared using the Nextera XT DNA Library Preparation Kit (Illumina Inc.) according to the manufacturers’ instructions. Paired-end sequencing was performed on the Illumina Hiseq 2500 platform. The raw reads were evaluated with FastQC v0.12.1, quality-filtered with fastp v1.3.3, and de novo assembled using SPAdes v4.3.0. The sequence type (ST), capsular (K), and lipopolysaccharide (O) antigen loci were determined using Kleborate v2.3.0. Genomic annotation was performed in parallel using Bakta v1.12.0 and Prokka v1.15.6. Bakta provided the primary gene coordinates and functional models, while Prokka was used as a secondary validator to examine open reading frames (ORFs) and identify potential unannotated determinants. Antimicrobial resistance genes were predicted using ABRicate v1.0.1 (CARD v4.0.1 database) and RGI v6.0.5 (‘Perfect’ and ‘Strict’ criteria) with a threshold of ≥ 80% identity and coverage. Outputs were then manually curated via NCBI BLAST. Virulence factors were annotated using ABRicate v1.0.1 against the VFDB database with ≥ 80% identity and coverage, and genome maps were rendered using Proksee. Integrons were detected and characterized using IntegronFinder v2.0. The genome sequence was deposited in GenBank under accession number JCBHFW000000000 (BioProject PRJNA1496901).

## Results and discussion

### Genomic typing and characterization of surface loci

The draft genome assembly of *K. pneumoniae* LC808 yielded a genome size of approximately 5.33 megabase pairs (Mbp), spanning 138 contigs with an N50 value of 256.3 kilobase pairs (Kbp). The genome exhibits a GC content of 57.5% and an average sequencing coverage of 30X (Supplementary Table S3). Genomic analysis confirmed that the LC808 isolate is a *K. pneumoniae* belonging to ST17 and carries the KL127 capsular locus and the O13 antigen. ST17 is globally distributed and frequently associated with multidrug resistance and severe human infections (Bahati and Maghembe 2025). Although ST17 has been identified in human, animal, and environmental samples, its occurrence in bovine mastitis is poorly documented (Huynh et al. 2020). In Asia, ST17 isolates have been found in cows with mastitis, healthy animals, and bulk tank milk (Wu et al. 2022; Wang et al. 2025; Kang et al. 2025; Adhyaru et al. 2026). In Brazil, *K. pneumoniae* ST17 has been identified in human clinical settings. This has been demonstrated by strains KpPi149 and KpPi150 (Nakamura-Silva et al. 2021), as well as by clinical isolates harboring *wzi93*-KL112 and producing SHV-2a (Andrade et al. 2018). This finding broadens our knowledge of the detection of this lineage ST17 in Brazil, including in cases of bovine mastitis. Nevertheless, our comparative analysis is strictly descriptive because no phylogenomic or genome-distance assessment was performed to infer direct evolutionary links. Further phylogenomic studies that include broader global ST17 datasets are required to establish these relationships.

### Characterization of the resistome

The LC808 genome exhibited a broad repertoire of acquired resistance genes (Supplementary Table S4), which is consistent with the MDR phenotype (Table 1). Four β-lactamase genes (*bla*_CTX−M−15_, *bla*_TEM−1_, *bla*_SHV−187_, and *bla*_OXA−1_) were identified. Phenotypic testing confirmed that isolate LC808 is an ESBL producer, consistent with the detection of *bla*_CTX−M−15_. The identification of ESBL determinants suggests the need for continuous herd-level monitoring (Zheng et al. 2022).

Additionally, genomic analysis revealed a complete class 1 integron region on contig NODE_40, carrying the *dfrA14* cassette, which confers trimethoprim resistance (Supplementary Table S5). Despite harboring the *catB3* gene, isolate LC808 displayed phenotypic susceptibility to chloramphenicol (Table 1), suggesting a silent resistance determinant likely resulting from promoter inactivity, transcriptional repression, or low in vitro expression levels (Graf et al. 2024). Regarding fosfomycin, the genome carried the *fosA6* gene, while the disk diffusion assay (200 µg fosfomycin / 50 µg G6P) yielded an inhibition zone diameter of 20 mm. No categorical susceptibility was assigned because CLSI disk diffusion interpretive criteria are restricted to urinary *Escherichia. coli* isolates. These results represent strain LC808’s overall resistome repertoire, but they do not imply specific plasmid co-transfer mechanisms because short-read draft assemblies limit contig linkage. Further long-read sequencing approaches are necessary to fully elucidate the mobilome architecture of this isolate.

**Table 1:** LC808 antibiotic susceptibility profile and resistance genes associated

Antibiotic	Susceptibility profile	AMR genes detected by WGS
Ampicillin (AMP)	R	*blaCTX-M-15*,* blaTEM-1*,* blaOXA-1*,* blaSHV-187*,* mdtQ*,* kpnGH*,* ompK37*,* acrAB-tolC*
Amoxicillin-clavulanate (AMC)	I	*blaCTX-M-15*,* blaOXA-1*,* kpnGH*,* acrAB-tolC*
Piperacillin-tazobactam (PPT)	I	*blaCTX-M-15*,* blaOXA-1*,* kpnGH*,* acrAB-tolC*
Cefazolin (CFZ)	R	*blaCTX-M-15*,* blaTEM-1*,* blaSHV-187*,* mdtQ*,* kpnEF*,* ompK37*,* acrAB-tolC*
Cefuroxime (CRX)	R	*blaCTX-M-15*,* blaTEM-1*,* blaSHV-187*,* mdtQ*,* kpnEF*,* ompK37*,* acrAB-tolC*
Cefotaxime (CTX)	R	*blaCTX-M-15*,* mdtQ*,* kpnEF*,* ompK37*,* acrAB-tolC*
Ceftazidime (CAZ)	R	*blaCTX-M-15*,* mdtQ*,* kpnEF*,* ompK37*,* acrAB-tolC*
Aztreonam (ATM)	R	*blaCTX-M-15*,* mdtQ*,* kpnEF*,* ompK37*,* acrAB-tolC*
Imipenem (IPM)	S	*-*
Meropenem (MER)	S	*-*
Ertapenem (ETP)	S	*-*
Gentamicin (GEN)	R	*aph(6)-Id*,* aph(3”)-Ib*,* aph(3’)-Ia*,* aac(3)-IIe*,* aac(6’)-Ib10*,* kpnEFGH*
Amikacin (AMI)	S	*aph(6)-Id*,* aph(3”)-Ib*,* aph(3’)-Ia*,* aac(3)-IIe*,* aac(6’)-Ib10*,* kpnEFGH*
Tetracycline (TET)	R	*tet(A)*,* kpnE*,* acrAB-tolC*,* oqxAB*
Minocycline (MIN)	I	*tet(A)*,* kpnE*,* acrAB-tolC*,* oqxAB*
Levofloxacin (LVX)	I	*qnrB17*,* oqxAB*,* kpnGH*,* acrAB-tolC*
Ciprofloxacin (CIP)	R	*qnrB17*,* oqxAB*,* kpnGH*,* acrAB-tolC*
Nalidixic acid (NAL)	I	*qnrB17*,* oqxAB*,* kpnGH*,* acrAB-tolC*
Trimethoprim-sulfamethoxazole (SXT)	R	*sul*,* dfrA14*
Trimethoprim (TRI)	R	*dfrA14*
Chloramphenicol (CHL)	S	*catB3*^**1**^, *acrAB-tolC*
Nitrofurantoin (NIT)	S	*oqxAB*
Fosfomycin (FOS)^**2**^	-	*FosA6*

1Discrepancies between detected resistance genes and phenotypic susceptibility may result from unexpressed, silent, or unpromoted genes

2There are no CLSI-validated cutoff values for disk diffusion testing in *Klebsiella pneumoniae*

Categories according to CLSI (2021): *S* susceptible, *I* intermediate, *R* resistant, *WGS* whole-genome sequencing, *AMR* resistance genotype

A descriptive comparison with Asian isolates of *K. pneumoniae* ST17 associated with bovine mastitis revealed that LC808 exhibited a distinct resistance profile. Wang et al. (2025), for example, identified resistance genes for β-lactams, aminoglycosides, quinolones, sulfonamides, and macrolides in China. Meanwhile, Kang et al. (2025) in South Korea reported limited resistance to ampicillin mediated by *bla*_SHV_. However, these comparisons should be interpreted with caution, given that discrepancies among published reports may stem from a lack of methodological standardization and differences in the phenotypic panels used for antimicrobial susceptibility testing (AST). Nevertheless, the identification of these determinants highlights the diverse resistance profiles circulating in dairy herds.

### Putative virulence associated and iron-acquisition determinants

The virulome of isolate LC808 included determinants associated with iron uptake (e.g., enterobactin, ferrienterobactin, and the *fecABCDEIR* operon), adhesion and biofilm formation (e.g., type 1 and type 3 fimbriae), and bacterial competition (e.g., the type VI secretion system) (Supplementary Table S6). Since no phenotypic assays were performed for adhesion, biofilm formation, or functional expression, these findings, which are based on in silico predictions, reflect genomic potential rather than proven intramammary adaptation.

The presence of multiple iron-acquisition systems may provide complementary mechanisms for iron uptake and may contribute to bacterial adaptation and persistence within the intramammary environment (Zheng et al. 2022; Bahati and Maghembe 2025). Further genomic analysis revealed that the complete *fecABCDEIR* operon is located at NODE_30, close to the transposable elements *tnpA* and *tnpB* (Figure S1). This suggests that the iron acquisition mechanism may be associated with a mobile genetic structure. However, the LC808 isolate lacks acquired determinants of lactose utilization, carrying only the intrinsic chromosomal. This contrasts with reports by Biggel et al. (2025) describing *K. pneumoniae* strains that carry the conserved *lac*_*aqc*_ e *fec* genes.

## Conclusion

Genomic characterization of isolate LC808 identified an ESBL-producing, multidrug-resistant *K. pneumoniae* ST17 strain from bovine mastitis milk in Brazil. The isolate carried the KL127 and O13 loci and exhibited a multidrug-resistant profile, including multiple clinically relevant resistance genes such as *bla*_CTX−M−15_. Identifying a class 1 integron that contains the *dfrA14* cassette confirms the presence of a structured resistance gene platform. These findings are based on a single clinical isolate and lack comparative sampling across the animal, food, and human sectors necessary to demonstrate cross-sector transmission. However, they document the presence of an ESBL-producing, multidrug-resistant ST17 strain in Brazilian dairy production. These results underscore the importance of genomic surveillance as a baseline in livestock settings within a One Health framework.

## Supplementary Information

Below is the link to the electronic supplementary material.

Supplementary Material 1

## Data Availability

The genome sequence was deposited in GenBank under accession number JCBHFW000000000 (BioProject PRJNA1496901).
