# Supplementary material for: Genomic characterization of a Brazilian multidrug-resistant Klebsiella pneumoniae ST17 isolate associated with bovine mastitis
Source: Vet Res Commun. 2026 Nov 11;51(1):50. doi: 10.1007/s11259-026-11622-5 (PMC13751694; doi:10.1007/s11259-026-11622-5)
Supplement: Supplementary file 1 [file 11259_2026_11622_MOESM1_ESM.docx]

**Genomic characterization of a Brazilian multidrug-resistant *Klebsiella pneumoniae* ST17 isolate associated with bovine mastitis**

Guilherme Castro Luz da Silva^1,2^ , ORCID: https://orcid.org/0009-0001-7806-5694

Rossiane de Moura Souza ^2^ , ORCID: https://orcid.org/0000-0001-9807-4291

Ana Carolina Paulo Vicente ^3^, ORCID: https://orcid.org/0000-0001-7086-2042

Sergio Mascarenhas Morgado ^3^, ORCID: https://orcid.org/0000-0003-4877-7639

Juliana Souza Alves ^4^, ORCID: https://orcid.org/0000-0001-9132-9554

Sílvia Conceição Reis Pereira Mello ^2^ , ORCID: https://orcid.org/0000-0002-5537-3563

Gustavo Luis de Paiva Anciens Ramos ^1^, ORCID: https://orcid.org/0000-0001-5631-9668

Alice Gonçalves Martins Gonzalez ^1*^ , ORCID: https://orcid.org/0000-0001-6789-502X

^1^ Fluminense Federal University, Faculty of Pharmacy, Department of Bromatology, Hygiene and Food Microbiology Laboratory, 24241-000, Brazil;

^2^ Agricultural Research Company of the State of Rio de Janeiro, State Centre for Research in Animal Health, Biotechnology Laboratory, 24120-191, Brazil;

^3^ Oswaldo Cruz Institute - FIOCRUZ - Rio de Janeiro, 21040-360, Brazil;

^4^ Federal University of Rio de Janeiro, Department of Natural Products and Foods - Rio de Janeiro, 21941-902, Brazil.

**SUPPLEMENTARY MATERIAL**

**Table S1.** Clinical metadata and case definition for bovine mastitis isolate LC808.

| **Parameter** | **Clinical Metadata** |
| --- | --- |
| Host species / Production | Bovine (Bos taurus), lactating dairy cow |
| Farm type / Location | Small-scale family dairy farm, Rio de Janeiro, Brazil |
| Sampling date | August 2022 |
| Clinical case definition | Presence of severe local inflammatory signs (mammary gland swelling, localized hyperthermia, severe pain on palpation) and marked milk abnormalities (milk clots with gross blood) |
| Clinical severity | Severe local clinical mastitis |
| Affected quarte | Right hind quarter |
| Antimicrobial treatment history | No antimicrobial therapy prior to sample collection |
| Concurrent pathogens | None detected (pure culture obtained on chromogenic media) |
| Clinical outcome | Full clinical recovery following post-sampling veterinary treatment protocol |

**Table S2.** Antimicrobial susceptibility testing (AST) profile, ESBL phenotypic detection, and epidemiological categorization of *Klebsiella pneumoniae* isolate LC808 recovered from bovine mastitis milk.

| **Antimicrobial Agent (µg)** | **Zone Diameter (mm)** | **CLSI Interpretation^1^** | **Epidemiological Terminology^2^** |
| --- | --- | --- | --- |
| β-LACTAMS |  |  |  |
| Ampicillin (10 µg) | 0 mm | Resistant | Not Susceptible |
| Amoxicillin-clavulanate (20 / 10 µg) | 15 mm | Intermediate | Not Susceptible |
| Piperacillin-tazobactam (100 / 10 µg) | 20 mm | Intermediate | Not Susceptible |
| Cefazolin (30 µg) | 0 mm | Resistant | Not Susceptible |
| Cefuroxime (30 µg) | 0 mm | Resistant | Not Susceptible |
| Cefotaxime (30 µg) | 17 mm | Resistant | Not Susceptible |
| Ceftazidime (30 µg) | 13 mm | Resistant | Not Susceptible |
| Aztreonam (30 µg) | 15 mm | Resistant | Not Susceptible |
| Imipenem (10 µg) | 25 mm | Susceptible | Susceptible |
| Meropenem (10 µg) | 27 mm | Susceptible | Susceptible |
| Ertapenem (10 µg) | 26 mm | Susceptible | Susceptible |
| AMINOGLYCOSIDES |  |  |  |
| Gentamicin (10 µg) | 0 mm | Resistant | Not Susceptible |
| Amikacin (10 µg) | 20 mm | Susceptible | Susceptible |
| TETRACYCLINES |  |  |  |
| Tetracycline (30 µg) | 0 mm | Resistant | Not Susceptible |
| Minocycline (30 µg) | 15 mm | Intermediate | Not Susceptible |
| QUINOLONES AND FLUOROQUINOLONES |  |  |  |
| Ciprofloxacin (5 µg) | 19 mm | Resistant | Not Susceptible |
| Levofloxacin (5 µg) | 20 mm | Intermediate | Not Susceptible |
| Nalidixic acid (30 µg) | 15 mm | Intermediate | Not Susceptible |
| FOLATE PATHWAY ANTAGONISTS |  |  |  |
| Trimethoprim-sulfamethoxazole (1.25 / 23.75 µg) | 0 mm | Resistant | Not Susceptible |
| Trimethoprim (5 µg) | 0 mm | Resistant | Not Susceptible |
| PHENICOLS |  |  |  |
| Chloramphenicol (30 µg) | 23 mm | Susceptible | Susceptible |
| NITROFURANS |  |  |  |
| Nitrofurantoin (300 µg) | 17 mm | Susceptible | Susceptible |
| FOSFOMYCINS |  |  |  |
| Fosfomycin (200 µg/G6P 50 µg)* | 20 mm | - | - |
| ESBL TEST^1^ |  |  |  |
| Ceftazidime (30 µg) | 13 mm | Resistant | - |
| Ceftazidime-clavulanate (30 / 10 µg) | 20 mm | ESBL-producing | - |
| Cefotaxime (30 µg) | 17 mm | Resistant |  |
| Cefotaxime-clavulanate (30 / 10 µg) | 23 mm | ESBL-producing | - |

* For fosfomycin, only the diameter of the inhibition zone (20 mm) is reported since the CLSI's established cut-off values for the disk diffusion test are limited to urinary *Escherichia coli* isolates.

^1^ CLSI (2021).

^2^ Magiorakos et al (2012).

**CLSI:** Clinical and Laboratory Standards Institute; **ESBL:** Extended-spectrum β-lactamase; **G6P**: glucose-6-phosphate.

**Table S3.** Genome assembly metrics and sequencing statistics for Klebsiella pneumoniae strain LC808.

| **Assembly statistics** | **Values** |
| --- | --- |
| Genome size | 5.329.845 Mb |
| Total ungapped length | 5.329.845 Mb |
| Number of contigs | 138 |
| Contig N50 | 256.3 kb |
| Contig L50 | 7 |
| GC percent | 57.5 |
| Genome coverage | 30x |

**pb**: base pair; **Kb**: thousands of bases; **Mb**: millions of bases; **GC**: guanine and cytosine.

**Table S4.** Antimicrobial resistance genes (ARGs) and resistance-associated determinants identified in the genome of *Klebsiella pneumoniae* isolate LC808.

| **Gene** | **Function** | **Coverage (%)** | **Identity (%)** | **Database** |
| --- | --- | --- | --- | --- |
| *qnrB17* | quinolone resistance protein | 99.85 | 99.12 | CARD |
| *tet(A)* | metal-tetracycline/H+ antiporter | 97.80 | 100.00 | CARD |
| *bla*SHV-187 | β-lactamase classic | 100.00 | 99.54 | CARD |
| *bla*CTX-M-15 | ESBL | 100.00 | 100.00 | CARD |
| *bla*TEM-1 | β-lactamase classic | 100.00 | 99.88 | CARD |
| *bla*OXA-1 | class D oxacillinase | 100.00 | 100.00 | CARD |
| *aac(3)-IIe* | AMEs | 100.00 | 99.53 | CARD |
| *aph(6)-Id* | AMEs | 100.00 | 99.88 | CARD |
| *aph(3'')-Ib* | AMEs | 100.00 | 99.75 | CARD |
| *aac(6')-Ib10* | AMEs | 96.08 | 99.66 | CARD |
| *aph(3')-Ia* | AMEs | 99.75 | 99.39 | CARD |
| **Gene** | **Function** | **Coverage (%)** | **Identity (%)** | **Database** |
| *sul2* | DHPS | 100.00 | 100.00 | CARD |
| *dfrA14* | DHFR | 100.00 | 100.00 | CARD |
| *catB3* | CAT | 100.00 | 100.00 | CARD |
| *fosA6* | glutathione S-transferase enzyme | 98.33 | 99.76 | CARD |
| *uhpT E350Q* | Hexose phosphate transporter (with mutation) | 100.00 | 95.03 | CARD |
| *marR* | AcrAB-TolC efflux complex regulatory protein | 100.00 | 93.33 | CARD |
| *marA* | AcrAB-TolC efflux complex transcriptional activator | 96.09 | 81.57 | CARD |
| *mdtC* | MDR efflux pump | 100.00 | 81.77 | CARD |
| *mdtB* | MDR efflux pump | 99.90 | 81.03 | CARD |
| *mdtQ* | MDR efflux pump | 100.00 | 99.58 | CARD |
| *baeS* | MdtABC System activator | 99.00 | 99.00 | CARD |
| *baeR* | MdtABC System Regulator | 100.00 | 93.33 | CARD |
| *oqxB* | RND-family efflux pump subunit | 100.00 | 98.45 | CARD |
| *oqxA* | RND-family efflux pump subunit | 100.00 | 99.15 | CARD |
| *arnT* | LPS Modification | 100.00 | 99.88 | CARD |
| *kpnG (emrA)* | Active efflux pump subunit | 100.00 | 99.23 | CARD |
| *kpnH* | Active efflux pump subunit | 99.94 | 84.42 | CARD |
| *kpnF* | Active efflux pump subunit | 100.00 | 100.00 | CARD |
| *kpnE* | Active efflux pump subunit | 100.00 | 99.45 | CARD |
| *ompK37* | Outer membrane porin | 100.00 | 99.11 | CARD |
| *ompA* | Outer membrane porin | 100.00 | 99.91 | CARD |
| *lptD* | LPS assembly and transport | 100.00 | 99.57 | CARD |
| *H-NS* | Repressor *acrE*, *mdtE* and *emrK* and multiple RND drugs. | 97.83 | 87.16 | CARD |
| *ramA* | Positive regulator of AcrAB-TolC and OmpF | 94.40 | 81.13 | CARD |
| *CRP* | Global transcription regulator | 100.00 | 87.84 | CARD |
| *acrA* | MDR efflux pump system | 99.75 | 97.91 | CARD |
| *acrB* | MDR efflux pump system | 99.75 | 83.91 | CARD |
| *TolC* | MDR efflux pump system | 98.00 | 97.00 | CARD |
| *eptB* | LPS Modification | 100.00 | 99.65 | CARD |

**CARD**: Comprehensive Antibiotic Resistance Database v.4.0.1 (Alcock et al., 2023);

**AME:** Aminoglycoside modifying enzyme; **CAT:** Chloramphenicol acetyltransferase; **DHFR**: dihydrofolate reductase enzyme; **DHPS**: dihydropteroate synthase enzyme; **ESBL:** Extended-spectrum β-lactamase; **LPS:** Lipopolysaccharide; **MDR**: Multidrug resistance.

**Table S5.** Structural characterization, genomic coordinates, and functional annotation of the complete class 1 integron identified on contig NODE_40 in *Klebsiella pneumoniae* LC808.

| **Feature / Element** | **Start (bp)** | **End (bp)** | **Strand** | **Predicted product** | **HMM model / Annotation** | **E-value / identity** |
| --- | --- | --- | --- | --- | --- | --- |
| Integrase gene (*intI1*) | 5,334 | 6,347 | +1 | class 1 integron integrase (*intI1*) | intersection_tyr_intI | 7.3e-26 |
| Recombination site (*attC*) | 4,625 | 4,710 | -1 | primary *attC* recombination site | attc_4 | 0.0052 |
| Resistance cassette (*dfrA14*) | 4,705 | 5,187 | -1 | dihydrofolate reductase | BLASTp match to VCW39271.1 | 100% identity (160/160 aa) |
| ORF (*mobC*) | 4,218 | 4,484 | -1 | plasmid mobilization relaxosome protein (MobC) | BLASTp match to KAB1871387.1 | 100% identity (88/88 aa) |

**ORF:** Open Reading Frame; **bp:** base pair; **HMM:** Hidden Markov Model; **aa:** amino acids;

**Table S6.** Virulence factor genes identified in the genome of *Klebsiella pneumoniae* isolate LC808.

| **Gene** | **Function** | **Coverage (%)** | **Identity (%)** | **Database** |
| --- | --- | --- | --- | --- |
| *tssG* | Type VI secretion system | 100.00 | 99.63 | VFDB |
| *tssF* | Type VI secretion system | 100.00 | 99.37 | VFDB |
| *tssH* | Type VI secretion system | 100.00 | 98.91 | VFDB |
| *tssM* | Type VI secretion system | 89.12 | 97.89 | VFDB |
| *tssD* | Type VI secretion system | 100.00 | 99.80 | VFDB |
| *tssL* | Type VI secretion system | 100.00 | 99.28 | VFDB |
| *tssK* | Type VI secretion system | 100.00 | 99.70 | VFDB |
| *tssC* | Type VI secretion system | 100.00 | 98.71 | VFDB |
| *tssB* | Type VI secretion system | 100.00 | 99.59 | VFDB |
| *tssJ* | Type VI secretion system | 100.00 | 99.63 | VFDB |
| *ompA* | Type VI secretion system | 100.00 | 83.75 | VFDB |
| *iutA* | Siderophore | 100.00 | 99.50 | VFDB |
| *iroE* | Siderophore | 100.00 | 99.04 | VFDB |
| *fecA* | Ferric-citrate transport | 98.00 | 99.00 | VFDB |
| *fecB* | Ferric-citrate transport | 94.00 | 88.00 | VFDB |
| *fecC* | Ferric-citrate transport | 96.00 | 95.00 | VFDB |
| *fecD* | Ferric-citrate transport | 99.00 | 99.00 | VFDB |
| *fecE* | Ferric-citrate transport | 93.00 | 93.00 | VFDB |
| *fecI* | Regulatory system | 98.00 | 98.00 | VFDB |
| *fecR* | Regulatory system | 88.00 | 89.00 | VFDB |
| *entD* | Siderophore | 100.00 | 99.36 | VFDB |
| *entF* | Siderophore | 100.00 | 99.36 | VFDB |
| *entS* | Siderophore | 100.00 | 98.87 | VFDB |
| *entC* | Siderophore | 100.00 | 99.49 | VFDB |
| *entE* | Siderophore | 100.00 | 99.44 | VFDB |
| *entB* | Siderophore | 100.00 | 98.71 | VFDB |
| *entA* | Siderophore | 100.00 | 83.75 | VFDB |
| *fes* | Siderophore | 100.00 | 99.42 | VFDB |
| *fepA* | Siderophore | 100.00 | 99.60 | VFDB |
| **Gene** | **Function** | **Coverage (%)** | **Identity (%)** | **Database** |
| *fepC* | Siderophore | 100.00 | 99.50 | VFDB |
| *fepG* | Siderophore | 100.00 | 99.60 | VFDB |
| *fepD* | Siderophore | 100.00 | 99.90 | VFDB |
| *fepB* | Siderophore | 100.00 | 99.38 | VFDB |
| *fimK* | Adhesion - Fimbriae | 100.00 | 99.29 | VFDB |
| *fimH* | Adhesion - Fimbriae | 99.67 | 99.01 | VFDB |
| *fimG* | Adhesion - Fimbriae | 100.00 | 99.59 | VFDB |
| *fimF* | Adhesion - Fimbriae | 100.00 | 99.43 | VFDB |
| *fimD* | Adhesion - Fimbriae | 100.00 | 99.58 | VFDB |
| *fimC* | Adhesion - Fimbriae | 100.00 | 99.59 | VFDB |
| *fimI* | Adhesion - Fimbriae | 100.00 | 99.63 | VFDB |
| *fimA* | Adhesion - Fimbriae | 100.00 | 99.82 | VFDB |
| *fimE* | Adhesion - Fimbriae | 100.00 | 99.34 | VFDB |
| *fimB* | Adhesion - Fimbriae | 100.00 | 99.34 | VFDB |
| *ykgK/ecpR* | Adhesion | 100.00 | 86.56 | VFDB |
| *yagZ/ecpA* | Adhesion | 99.32 | 89.90 | VFDB |
| *yagY/ecpB* | Adhesion | 100.00 | 87.59 | VFDB |
| *yagX/ecpC* | Adhesion | 99.96 | 87.42 | VFDB |
| *yagW/ecpD* | Adhesion | 100.00 | 88.44 | VFDB |
| *yagV/ecpE* | Adhesion | 100.00 | 84.81 | VFDB |
| *mrkA* | Biofilm - Fimbriae | 100.00 | 99.34 | VFDB |
| *mrkB* | Biofilm - Fimbriae | 100.00 | 100.00 | VFDB |
| *mrkC* | Biofilm - Fimbriae | 100.00 | 99.60 | VFDB |
| *mrkD* | Biofilm - Fimbriae | 100.00 | 99.90 | VFDB |
| *mrkF* | Biofilm - Fimbriae | 100.00 | 99.69 | VFDB |
| *mrkJ* | Biofilm - Fimbriae | 100.00 | 99.86 | VFDB |
| *mrkI* | Biofilm - Fimbriae | 100.00 | 99.30 | VFDB |
| *mrkH* | Biofilm - Fimbriae | 100.00 | 99.57 | VFDB |
| *acrB* | Adaptation to the environment | 100.00 | 99.78 | VFDB |
| *acrA* | Adaptation to the environment | 100.00 | 99.33 | VFDB |
| *rpoS* | Regulation | 99.80 | 86.91 | VFDB |
| *fur* | Regulation | 96.47 | 83.98 | VFDB |
| *ugd* | Capsule - immune modulation | 100.00 | 96.49 | VFDB |
| *galF* | Capsule - immune modulation | 100.00 | 98.88 | VFDB |
| *gndA* | Capsule - immune modulation | 100.00 | 92.68 | VFDB |
| KP1_RS17355 | Capsule - immune modulation | 99.84 | 95.39 | VFDB |
| *wzi* (KP1_RS1734) | Capsule - immune modulation | 100.00 | 87.22 | VFDB |
| KP1_RS17340 | Capsule - immune modulation | 99.29 | 80.39 | VFDB |
| *rcsA* | Regulation | 100.00 | 100.00 | VFDB |
| *rcsB* | Regulation | 100.00 | 99.85 | VFDB |

**VFDB:** Virulence Factor Database v.1.0.1 (Chen et al., 2005);

**Figure S1.** Genomic architecture of the ferric-citrate iron-acquisition cluster (*fecABCDEIR*) on contig NODE_30 of Klebsiella pneumoniae LC808, rendered using Proksee.

Green arrows denote annotated open reading frames (ORFs) according to Bakta and Prokka. The *fec* operon is directly flanked by transposable elements (*tnpA* upstream and *tnpB* downstream), indicating association with a mobile genetic structure.

**REFERENCES**

Alcock BP, Huynh W, Chalil R, Smith KW, Raphenya AR, Wlodarski MA, Edalatmand A, Petkau A, Syed SA, Tsang KK, Baker SJC, Dave M, McCarthy MC, Mukiri KM, Nasir JA, Golbon B, Imtiaz H, Jiang X, Kaur K, Kwong M, Liang ZC, Niu KC, Shan P, Yang JYJ, Gray KL, Hoad GR, Jia B, Bhando T, Carfrae LA, Farha MA, French S, Gordzevich R, Rachwalski K, Tu MM, Bordeleau E, Dooley D, Griffiths E, Zubyk HL, Brown ED, Maguire F, Beiko RG, Hsiao WWL, Brinkman FSL, Van Domselaar G, McArthur AG. 2023. CARD 2023: expanded curation, support for machine learning, and resistome prediction at the Comprehensive Antibiotic Resistance Database. Nucleic Acids Research, 51(1 D), D690–D699. https://doi.org/10.1093/nar/gkac920.

Chen, L., Yang, J., Yu, J., Yao, Z., Sun, L., Shen, Y., & Jin, Q. (2005). VFDB: A reference database for bacterial virulence factors. Nucleic Acids Research, 33(DATABASE ISS.), 325–328. https://doi.org/10.1093/nar/gki008.

CLSI, Clinical and Laboratory Standards Institute. Performance Standards for Antimicrobial Susceptibility Testing. 32th. ed. USA: CLSI supplement M100, 2021.

Magiorakos, A.-P., Srinivasan, A., Carey, R. B., Carmeli, Y., Falagas, M. E., Giske, C. G., Harbarth, S., Hindler, J. F., Kahlmeter, G., Olsson-Liljequist, B., Paterson, D. L., Rice, L. B., Stelling, J., Struelens, M. J., Vatopoulos, A., Weber, J. T., & Monnet, D. L. (2012). Multidrug-resistant, extensively drug-resistant and pandrug-resistant bacteria: An international expert proposal for interim standard definitions for acquired resistance. Clinical Microbiology and Infection, 18(3), 268–281. https://doi.org/10.1111/j.1469-0691.2011.03570.x.

Schwengers, O., Jelonek, L., Dieckmann, M. A., Beyvers, S., Bloom, J., & Goesmann, A. (2021). Bakta: Rapid and standardized annotation of bacterial genomes via alignment-free sequence identification. Microbial Genomics, 7(11). https://doi.org/10.1099/MGEN.0.000685.

Seemann, T. (2014). Prokka: Rapid prokaryotic genome annotation. Bioinformatics, 30(14), 2068–2069. https://doi.org/10.1093/bioinformatics/btu153.
